# The potential role of RNA N6-methyladenosine in Cancer progression

**DOI:** 10.1186/s12943-020-01204-7

**Published:** 2020-05-12

**Authors:** Tianyi Wang, Shan Kong, Mei Tao, Shaoqing Ju

**Affiliations:** 1 Department of Laboratory Medicine, Affiliated Hospital of Nantong University, NO.20, Xisi Road, Nantong, 226001 Jiangsu China; 2 Research Center of Clinical Medicine, Affiliated Hospital of Nantong University, NO.20, Xisi Road, Nantong, 226001 Jiangsu China; 3 School of Public Health, Nantong University, NO 9, Seyuan Road, Nantong, 226019 Jiangsu China

**Keywords:** N6-methyladenosine (m6A), Molecular mechanisms, Cancer progression

## Abstract

N6-methyladenosine (m6A) is considered the most common, abundant, and conserved internal transcript modification, especially in eukaryotic messenger RNA (mRNA). m6A is installed by m6A methyltransferases (METTL3/14, WTAP, RBM15/15B, VIRMA and ZC3H13, termed “writers”), removed by demethylases (FTO, ALKBH5, and ALKBH3, termed “erasers”), and recognized by m6A-binding proteins (YTHDC1/2, YTHDF1/2/3, IGF2BP1/2/3, HNRNP, and eIF3, termed “readers”). Accumulating evidence suggests that m6A RNA methylation greatly impacts RNA metabolism and is involved in the pathogenesis of many kinds of diseases, including cancers. In this review, we focus on the physiological functions of m6A modification and its related regulators, as well as on the potential biological roles of these elements in human tumors.

## Introduction

More than 160 chemically distinct RNA modifications have been identified, generating a new field known as “epitranscriptomics” [1]. N6-methyladenosine (m6A) was first discovered in eukaryotic messenger RNA (mRNA) [2, 3] and viral nuclear RNA [4, 5] in the 1970s and has been identified as one of the most common and abundant RNA modifications. However, research on this modification has been hindered, and its biological function remains largely unknown due to the lack of methods to define its location in RNA. It was not until 2011 that fat mass and obesity-associated protein (FTO) was identified as an m6A mRNA demethylase, establishing the view of m6A as a reversible modification [6] and making it a popular research focus. In 2012, two groups independently reported high-throughput sequencing of m6A at the whole transcriptome level [7, 8]. The development of m6A antibody affinity enrichment combined with high-throughput m6A sequencing and methylated RNA m6A immunoprecipitation sequencing (MeRIP-m6A-seq) provided a technical foundation for further research on m6A.

Estimates suggest that more than 7000 coding and 300 noncoding RNAs contain m6A and that 0.1–0.4% of the total adenine nucleotide content is methylated in mammalian transcripts [7–9]. In addition, a consensus sequence, “RRACH” ([G > A]m6AC[U > A > C]) [7–9] has been identified, and m6A is enriched in 3′ UTRs, near stop codons in mRNAs, or near the last exon in noncoding RNA [7, 8]. Similar to DNA methylation, m6A modification is reversible and catalyzed by corresponding enzymes, namely, “writers,” “erasers,” and “readers.” Studies have shown that aberrancies in m6A are associated with a variety of diseases, such as type 2 diabetes mellitus (DM2) [10] and cancers. This review focuses on the potential role of m6A in cancer progression.

## m6A writers, erasers, and readers

**Fig. 1:**
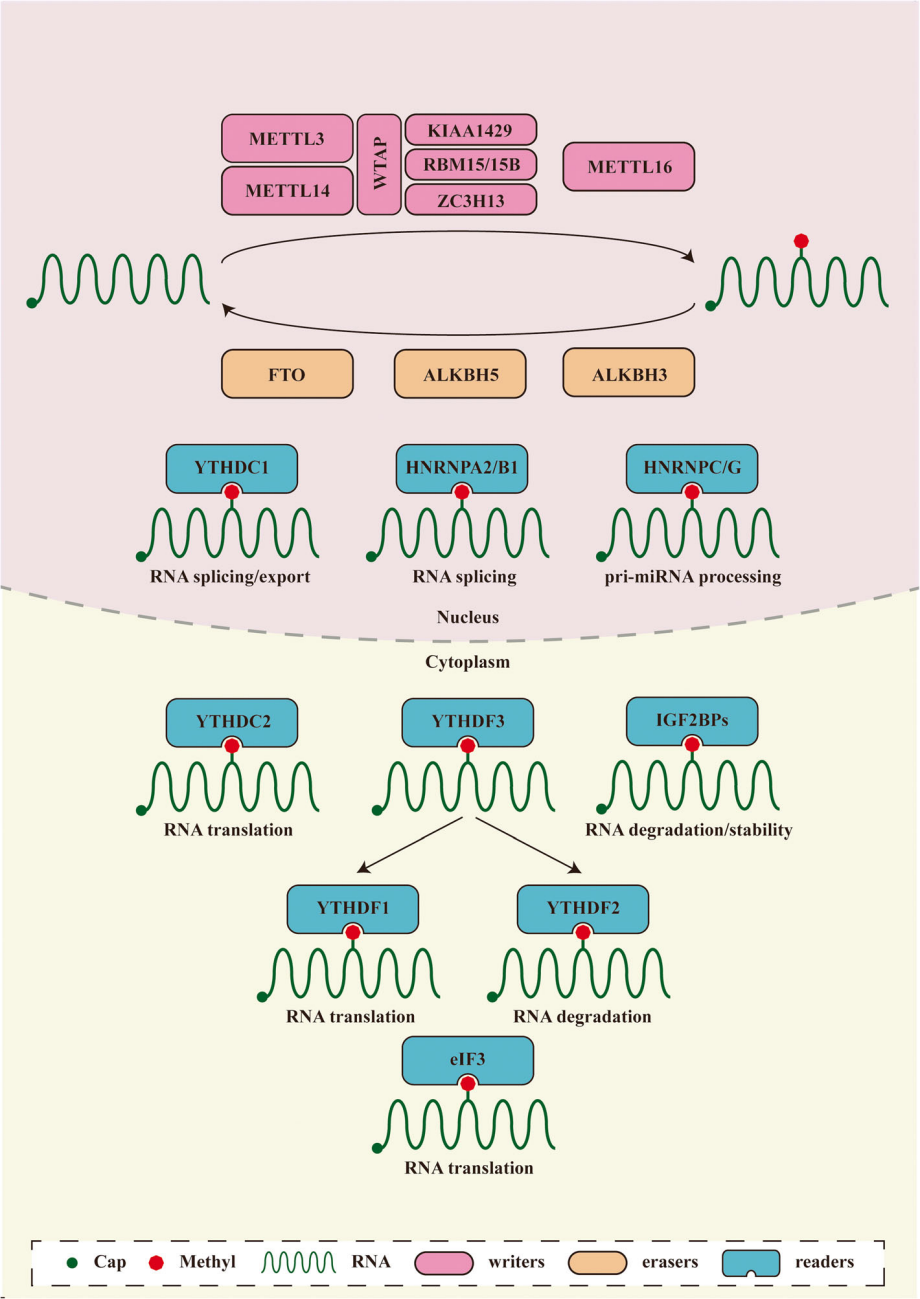
The molecular mechanism of m6A mutation. m6A is installed by “writers” (METTL3/14, WTAP, RBM15/15B, VIRMA and ZC3H13), removed by “erasers” (FTO, ALKBH5, and ALKBH3, termed “erasers”), and recognized by “readers” (YTHDC1/2, YTHDF1/2/3, IGF2BP1/2/3, HNRNP, and eIF3, termed “readers”)

m6A writers, erasers and readers are proteins that can install, remove, or recognize, respectively, m6A on mRNAs or noncoding RNAs. These proteins play an important biological role in m6A modifications (Table 1, Fig. 1).

**Table 1:**
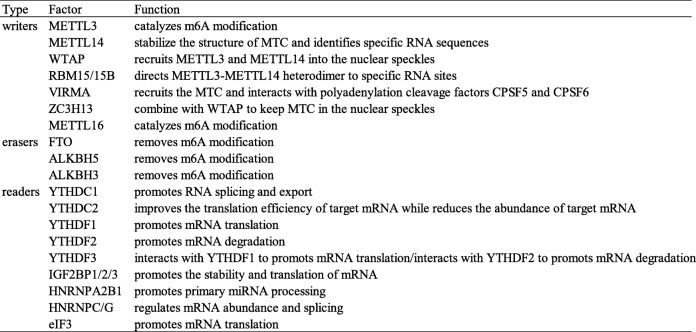
The functions of m6A enzymes in RNA metabolism

### Methyltransferases/writers

Installation of m6A is catalyzed by a methyltransferase complex (MTC) composed of several proteins [11]. Methyltransferase-like protein 3 (METTL3) is an S-adenosyl methionine (SAM)-binding protein; METTL3 is the most important component of the m6A MTC and is highly conserved in eukaryotes from yeast to humans [12]. Methyltransferase-like protein 14 (METTL14) is another active component of the m6A MTC. METTL3 and METTL14 are colocalized in nuclear speckles and form stable complexes in a 1:1 ratio [13]. However, only METTL3 acts as a catalyst, with an internal SAM-binding domain that catalyzes the transfer of methyl groups in SAM to adenine bases in RNA, producing S-adenosyl homocysteine (SAH), whereas METTL14 primarily acts to stabilize the structure of MTC and identifies specific RNA sequences (“RRACH”) as catalytic substrates [14–17]. WT1-associated protein (WTAP) has no catalytic function and promotes m6A installation mainly by recruiting METTL3 and METTL14 into nuclear speckles [18]. RNA-binding motif protein 15 (RBM15) and RNA-binding motif protein 15B (RBM15B) also have no catalytic function, but they can bind METTL3 and WTAP and direct these two proteins to specific RNA sites for m6A modification [19, 20]. Vir-like m6A methyltransferase associated (VIRMA/KIAA1429) preemptively recruits the MTC and mediates methylation of adenine bases near the 3′ UTR and stop codon regions in mRNA; it also interacts with cleavage and polyadenylation specificity factor subunit 5 (CPSF5) and cleavage and polyadenylation specificity factor subunit 6 (CPSF6) [21]. Additionally, 80% of the protein sequence of zinc finger CCCH-type containing 13 (ZC3H13) are low-complexity (LC) domains, which may have effects on the target nuclear speckles. After ZC3H13 interacts with WTAP, MTC is retained in nuclear speckles via its LC domains, which improves its catalytic function [20, 22]. Unlike METTL3, the other components of the catalytic complexes lack RNA methyltransferase activity.

Methyltransferase-like protein 16 (METTL16) is a newly discovered independent RNA methyltransferase. It can catalyze m6A installation in the 3′ UTR in mRNA and on A43 of U6 small nuclear RNA. A43 is located in a basic “ACAGAGA” box of U6. METTL16 binds to La protein, La-related protein 7 (LARP7) and methylphosphate capping enzyme (MEPCE) and installs U6-m6A43 on oligonucleotide transcripts during the early stages of U6 small nuclear ribonucleoprotein (snRNP) biogenesis. During splicing, the base pair forms a 5′ splice site in the associated pre-mRNA, suggesting that METTL16 plays an important role in splicing regulation [23, 24].

### Demethylases/erasers

The first m6A demethylase, FTO, was discovered in 2011 [6]. Soon after, in 2013, the second RNA demethylase, AlkB homolog 5 (ALKBH5), was discovered [25]. These findings fully indicate that m6A installation is a reversible process. FTO and ALKBH5 belong to the alpha-ketoglutarate-dependent dioxygenase family and catalyze demethylation of m6A in an Fe (II) and α-ketoglutaric acid-dependent manner. First, they oxidize m6A to form N6-hydroxymethyladenosine (hm6A) and then convert the hm6A to N6-formyladenosine (f6A). Finally, f6A is converted to adenosine (A), completing the demethylation process. In addition, recent studies have identified another m6A demethylase, AlkB homolog 3 (ALKBH3), which may complete the demethylation process through the above mechanism [26].

### Readers

**Fig. 2:**
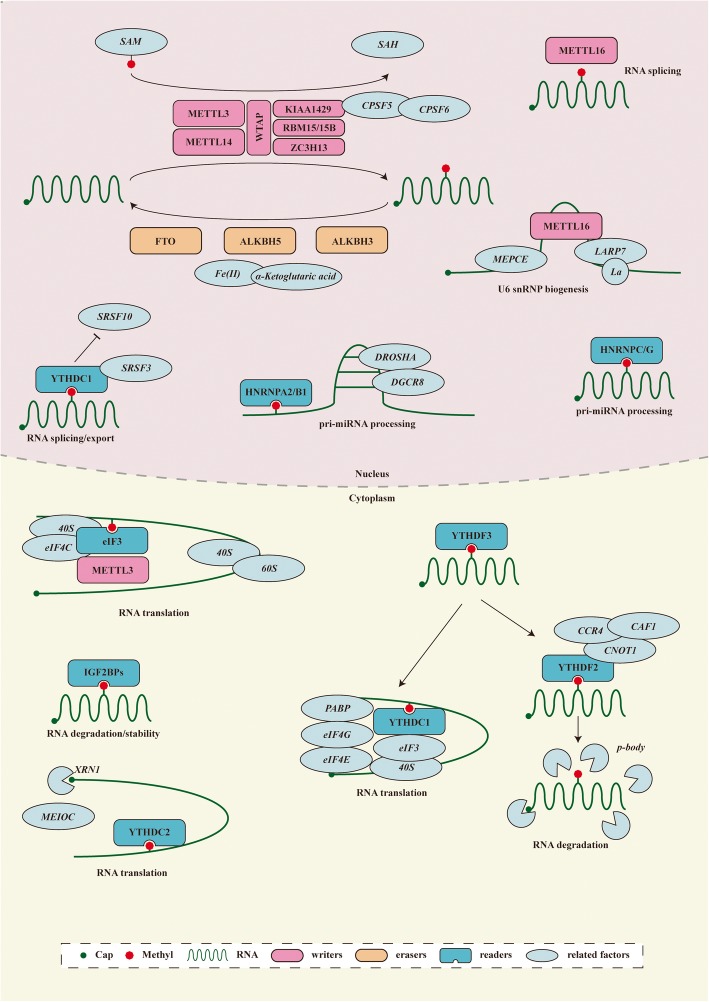
The detailed molecular mechanism of m6A enzymes. The “writers”, “erasers” and “readers” relay on various related factors install, remove and recognize m6A mutation and affect RNA metabolic processes, including RNA splicing, export, translation, degradation and so on

The functional interaction between m6A writers and erasers determines the dynamic and reversible regulation of m6A modification. However, for different downstream biological functions, m6A must be identified by various readers (Fig. 2).Members of the YT521-B homology (YTH) domain family, including YTH domain family protein 1(YTHDF1), YTH domain family protein 2 (YTHDF2), YTH domain family protein 3 (YTHDF3), YTH domain containing 1(YTHDC1), and YTH domain containing 2 (YTHDC2), have conserved m6A-binding domains that can bind to RNA containing m6A, and these molecules are the most important readers [27]. YTHDF2 was the first m6A reader to be identified. It can identify specific m6A sites via its C-terminal region, and its N-terminal region binds to the SH domain of CCR4-NOT transcription complex subunit 1 (CNOT1), thus directly recruiting the CCR4-NOT deadenylase complex and eventually transporting RNA to the processing body (P-body) to accelerate the degradation of m6A-modified RNA [28]. In contrast to YTHDF2, YTHDF1 can bind to m6A sites around stop codons in mRNA, and the translation initiation complex, including eukaryotic translation initiation factor 3 (eIF3), eukaryotic translation initiation factor 4E (eIF4E), eukaryotic translation initiation factor 4G (eIF4G), poly(A) binding protein (PABP), and the 40S ribosomal subunit, can be recruited to promote the translation of the target RNA [29]. YTHDF3 is currently considered to play a synergistic role, cooperating with YTHDF1 to promote the translation or accelerating the degradation of the target RNA through direct interaction with YTHDF2 [30, 31]. YTHDC1 promotes exon inclusion in the nucleus by recruiting serine- and arginine-rich splicing factor 3 (SRSF3) or antagonizing serine- and arginine-rich splicing factor 10 (SRSF10) [32]. YTHDC1 plays a synergistic role with nuclear RNA export factor 1 (NXF1) and the three prime repair exonuclease (TREX) mRNA export complex by interacting with SRSF3, thus promoting the export of m6A-methylated mRNAs from the nucleus [33, 34]. In addition, YTHDC2 can interact with meiosis-specific coiled-coil domain (MEIOC) and 5′-3′ exoribonuclease 1 (XRN1) after identifying m6A, thus improving the translation efficiency of the target mRNA while reducing its abundance [35, 36].

In addition to members of the YTH domain family, other proteins act as m6A readers. For example, members of the heterogeneous nuclear ribonucleoprotein (HNRNP) family also play an important role. HNRNPA2/B1 can recognize m6A on a subset of primary microRNA (pri-miRNA) transcripts and interact with drosha ribonuclease III (DROSHA) and DiGeorge syndrome critical region 8 (DGCR8), thus facilitating pri-miRNA processing [37]. In addition, m6A affects the secondary structure of RNA, whereas HNRNPC and HNRNPG can regulate mRNA abundance and splicing after recognizing m6A. This phenomenon is called the “m6A switch” [38, 39]. In addition, insulin-like growth factor 2 mRNA-binding proteins (IGF2BPs, including IGF2BP1/2/3) can identify m6A and promote mRNA stability and translation in an m6A-dependent manner [40]. However, the detailed mechanism is unclear. m6A in the 5′ UTR region of transcripts can also directly recruit eIF3, which can recruit the 43S ribosomal preinitiation complex to promote cap-independent translation [41]. Cytoplasmic METTL3 promotes cap-dependent translation in specific mRNA subsets by directly recruiting eukaryotic translation initiation factor 3H (eIF3H) [42].

### m6A modification and noncoding RNAs

Although most studies regarding m6A modification are focused on mRNA, some recent studies have shown that the m6A modification and its related regulatory factors also have biological effects on noncoding RNA. Noncoding RNAs include mainly miRNAs, long noncoding RNAs (lncRNAs), and circular RNAs (circRNAs) [43, 44]. m6A modification not only affects the cleavage, transport, stability, and degradation of noncoding RNAs but also regulates the function of biological cells by affecting the expression of noncoding RNAs [45, 46]. miRNAs belong to a small class of noncoding RNAs that are endogenous coding short RNAs (~ 22 nt) produced by the original transcript (pri-miRNAs) transcribed by RNA polymerase II/III [47, 48]. As mentioned above, pri-miRNAs can be further treated by DGCR8 and DROSHA, while pri-miRNAs modified by m6A installation catalyzed by the MTC can be recognized by HNRNPA2/B1 to promote the binding of DGCR8 and DROSHA, thus promoting the pri-miRNA maturation process of [49]. Through matrix-assisted laser desorption/ionization time-of-flight mass spectrometry (MALDI-TOF MS), Konno et al. found that miR-17-5p, miR-21-5p, miR-200c-3p, and miR-let-7a-5p contain m6A methylation sites in pancreatic cancer. In addition, although the expression of these miRNAs does not differ significantly in cancer tissues compared with matched normal tissues, their methylation levels are significantly increased. More importantly, the methylation level of serum miR-17-5p can distinguish early pancreatic cancer patients from healthy controls with high sensitivity and specificity, even exceeding those of traditional biomarkers such as CEA and CA19–9 [50]. lncRNAs are transcripts of more than 200 nucleotides in length that have no protein-coding function [51]. lncRNAs modified by m6A may also regulate biological processes. The effect of m6A modification on lncRNAs may involve a variety of regulatory mechanisms. On the one hand, m6A modification may regulate the functions of lncRNAs by providing binding sites for m6A reader proteins or by regulating the structure of local RNA to induce the binding of RNA-binding proteins (RBPs). On the other hand, m6A modification may regulate the relationship between RNA-DNA and specific DNA sites by affecting the triple helix structure of lncRNA. Studies have shown that METTL3 and RBM15/15B can mediate m6A modification of lncRNA-XIST. In addition, YTHDC1 recognition of the m6A modification site on lncRNA-XIST is also necessary for the basic silencing functions of lncRNA-XIST [19]. CircRNAs were first discovered in an RNA virus in the 1970s. CircRNAs belong to the noncoding RNA family, do not contain 3′ and 5′ ends, are usually produced by reverse splicing of pre-mRNA, and exist in a circularized form [52, 53]. Researchers have found that circRNAs, especially exon-derived circRNAs, can be modified by m6A methylation and that methylated circRNAs have protein-coding ability. A study showed that the m6A motif “RRACH” is highly expressed in circRNAs and that a single m6A site can completely drive translation initiation [54]. In addition, m6A modification of circRNAs is regulated by METTL3/14 and FTO. Furthermore, m6A modification can promote circRNA translation by mediating eIF4G2 and the binding protein YTHDF3 [55].

## Experimental methods for analyzing m6A modification

The total m6A content in RNA samples can be measured by several methods, including two-dimensional thin layer chromatography [56, 57], dotblotting [6], and high-performance liquid chromatography-tandem mass spectrometry (HPLC-MS/MS) [6, 25]. However, these methods can be used only for quantitative purposes, not for specific localization of the m6A modification site. The range distribution of the m6A-modified transcriptome was unknown until the introduction of MeRIP-m6A-seq in 2012 [7, 8]. Since then, this novel method has aroused attention and interest due to its accuracy and repeatability. In MeRIP-m6A-seq, mRNA is first cleaved into small fragments of approximately 100 nt, immunoprecipitated with a specific antibody against m6A, and finally subjected to high-throughput sequencing. Because the small RNA fragment targeted by MeRIP-m6A-seq has a resolution of approximately 100 nt, single-nucleotide variations at the methylated site cannot be accurately detected [7, 58]. Photo-cross-linking-assisted m6A sequencing (PA-m6A-seq) and site-specific cleavage and radioactive labeling followed by ligation-assisted extraction and thin layer chromatography (SCARLET) have a higher resolution than Me-RIP-m6A-seq [59, 60]. Currently, a new method called m6A individual nucleotide resolution crosslinking immunoprecipitation (miCLIP) symbolizes a large step forward in this field. miCLIP can accurately detect the position of m6A, which is very important for mapping the exact position of m6A-modified residues [61]. In addition, Kenno et al. proposed a new technique that uses matrix-assisted laser desorption/ ionization time of flight mass spectrometry (MALDI-TOF-MS) to specifically detect m6A mutations in related miRNA. The target miRNAs are purified by magnetic beads with bound complementary oligonucleotides and analyzed by MALDI-TOF-MS. Methylated nucleotides can be detected as a peak A + 14 Da from predicted non-methylated peaks and the methylation site can be further confirmed by derivatization of nucleotides [50].

## Databases for m6A modification analysis

Currently, m6A modifications can be analyzed by some available databases, including WHISTLE, DRUM, BERMP, PseDNC, MeT-DB, RMBase, m6AVar, m6AViewer, RNAMethPre, and SRAMP [62–71]. These databases can predict and analyze m6A modification sites, SNPs, RBPs, and RNA splicing patterns for different genes, cell lines, or diseases by using different computational methods to associate existing gene expression, protein expression, RNA methylation, disease, or cell line databases. The user-friendly and intuitive interface enables more convenient acquisition of data, which can be used in scientific research (Table 2).

**Table 2:**
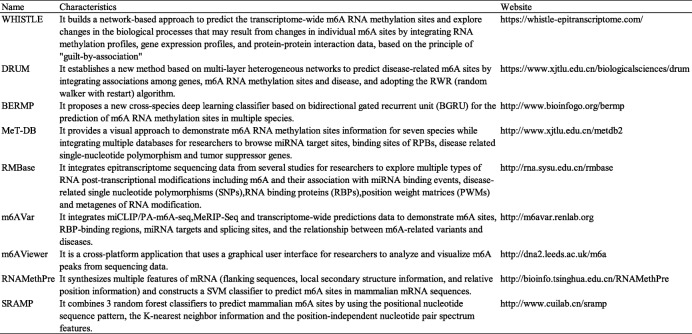
The details of databases for m6A modification analysis

## m6A modification and cancer

**Fig. 3:**
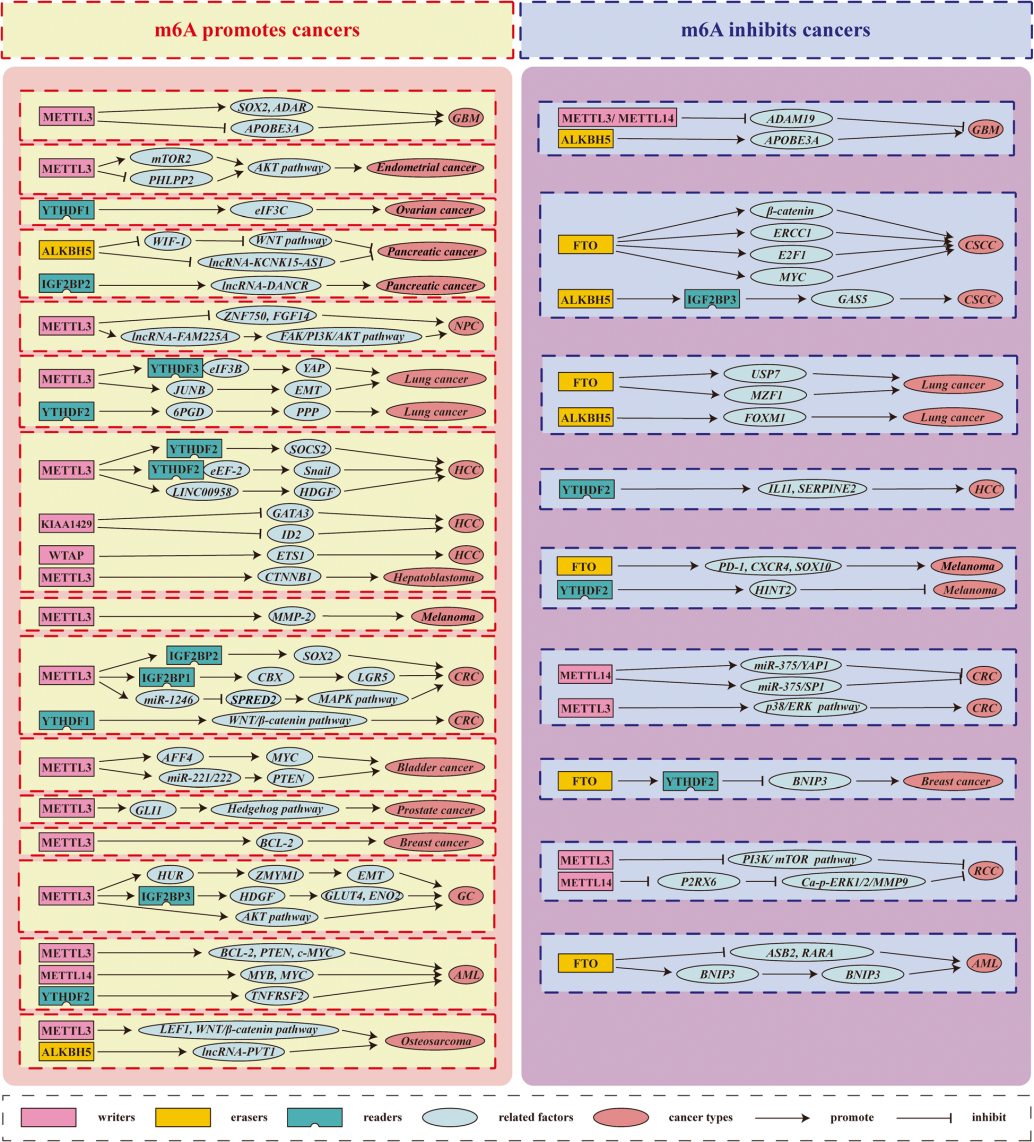
The potential roles of m6A in cancer progression. The potential role of m6A in cancer progression is reflected in the regulation of tumor-associated gene expression. m6A modification promotes cancer progression by enhancing oncogene expression and inhibiting tumor suppressor gene expression. m6A modification hinders cancer progression by inhibiting oncogene expression and enhancing tumor suppressor gene expression

Many studies have shown that m6A modification plays an important role in various cancers, often via the actions of writers that catalyze m6A modifications in the mRNA of oncogenes or tumor suppressor genes and then the actions of readers that recognize these marks through a series of molecular biological effects, in turn upregulating oncogene expression or downregulating tumor suppressor gene expression. Conversely, modification can occur via erasers that remove m6A from the mRNA of oncogenes or tumor suppressor genes, preventing recognition by readers from playing its appropriate molecular biological role, thus upregulating oncogene expression or downregulating tumor suppressor gene expression (Table 3, Fig. 3).

**Table 3:**
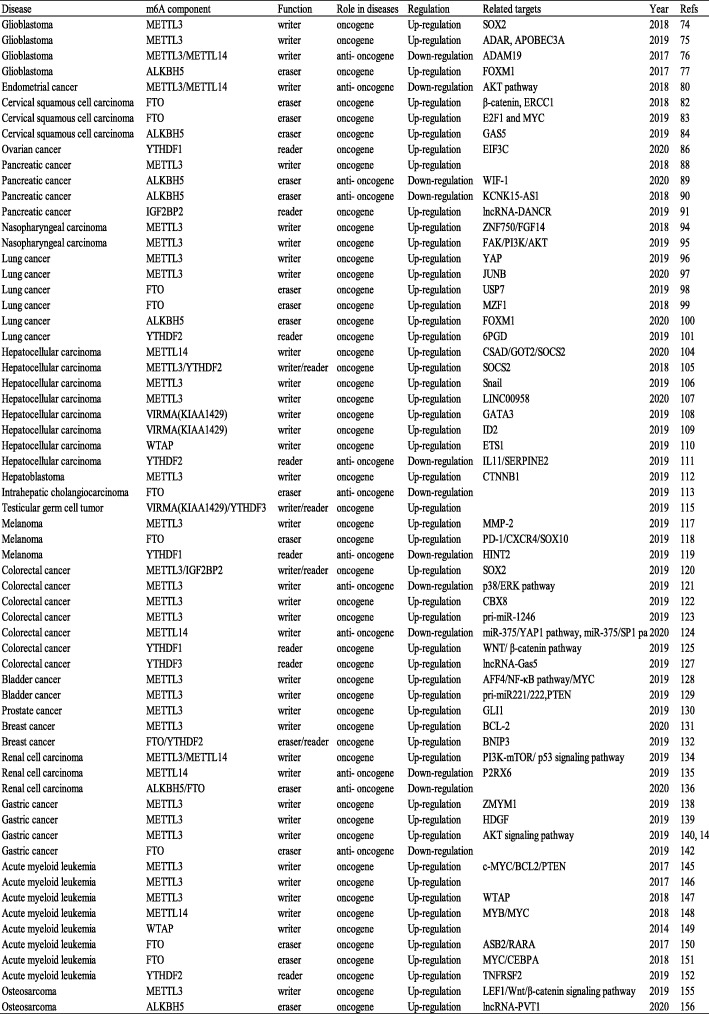
The roles of m6A enzymes in cancer progression

### Glioblastoma (GBM)

Among the various types of primary tumors, GBM is a fatal cancer that is considered incurable. Glioblastoma stem cells (GSCs) are considered a new therapeutic target for GBM [72]. In one study, 904 gliomas were divided into two subgroups, and differential expression of m6A regulatory factors was detected by cluster analysis. The researchers established a risk assessment model based on the expression of m6A regulatory factors to predict the clinicopathological features, WHO grade, mutation frequency, and epithelial-mesenchymal transition (EMT) of gliomas [73]. METTL3 promotes mRNA methylation and enhances the stability of SRY-box transcription factor 2 (SOX2), increases SOX2 protein expression, and promotes the maintenance and radiation resistance of glioma stem-like cells [74]. Additionally, METTL3 alters A-to-I and C-to-U RNA editing events by differentially regulating the RNA editing enzymes adenosine deaminase RNA specific (ADAR) and apolipoprotein B mRNA editing enzyme catalytic subunit 3A (APOBEC3A). Similar to protein-coding genes, long intergenic noncoding RNAs (lincRNAs) with m6A marks exhibit high METTL3-dependent expression. In summary, METTL3 plays a crucial role in many steps of RNA processing and coordinates the oncogenic pathway in GSCs [75]. In addition, studies have shown that METTL3 or METTL14 is beneficial to preventing GSC self-renewal and tumorigenicity and that ADAM metallopeptidase domain 19 (ADAM19) may be the target gene [76]. However, ALKBH5 was found to be highly expressed in GSCs, and silencing ALKBH5 inhibited the proliferation of GSCs. m6A-seq analysis performed by a comprehensive transcriptional research group showed that the expression of some ALKBH5 target genes, including forkhead box m1 (FOXM1), was altered. ALKBH5 demethylated FOXM1 mRNA, thus increasing FOXM1 expression. In addition, FOXM1 antisense noncoding RNA (FOXM1-AS) promoted the interaction between ALKBH5 and FOXM1 transcripts. Depleting ALKBH5 and FOXM1-AS abolished the tumorigenicity of GSCs through the FOXM1 axis [77]. Furthermore, Malacrida et al. found that the imidazobenzoxazin-5-thione MV1035 significantly inhibited ALKBH5, thereby reducing GBM invasiveness [78].

### Female reproductive system cancers

Endometrial cancer accounts for more than 70,000 deaths among women worldwide annually, and the high disease mortality makes endometrial cancer an important women’s health consideration [79]. m6A methylation was found to be decreased in approximately 70% of endometrial tumors, probably due to METTL14 mutation or a decrease in METTL3 expression. These two changes led to proliferation and tumorigenicity of endometrial cancer cells, probably due to activation of the AKT pathway. The decrease in m6A methylation decreased the expression of the negative AKT regulator PHLPP2 and increased the expression of the positive AKT regulator mTORC2 [80].

Cervical cancer is one of the most prevalent gynecological malignancies worldwide, with poor prognosis, and over 90% of cervical cancer cases are cervical squamous cell carcinoma (CSCC) [81]. The mRNA level of FTO in CSCC tissues was found to be higher than that in the corresponding paracancerous tissues. FTO downregulates the level of m6A in β-catenin gene transcripts and increases the expression of β-catenin. It also increases the expression of ERCC excision repair 1 (ERCC1), thus playing an important role in chemo- and radioresistance, which has potential clinical significance for the treatment of CSCC [82]. Additionally, interaction of FTO with E2F transcription factor 1 (E2F1) and MYC transcripts reduces m6A modification on the corresponding mRNA and enhances the translation efficiency of the mRNA, which is critical to the regulation of cervical cancer cell proliferation and migration [83]. In addition, researchers found low expression of lncRNA-GAS5-AS1 in cervical cancer cells; this lncRNA has been shown to interact with the tumor suppressor GAS5 and increase its stability by reducing ALKBH5-mediated m6A modification of GAS5 mRNA. In addition, the authors found that m6A-mediated GAS5 mRNA degradation was dependent on the pathway of the m6A reader protein YTHDF2 [84].

Ovarian cancer is the fifth leading cause of cancer-related death in women worldwide and has the highest mortality rate among all gynecologic cancers [85]. Via a multigroup analysis of ovarian cancer, Liu et al. used identified that YTHDF1 increased eIF3C translation in an m6A-dependent manner by binding to m6A-modified eIF3C mRNA while promoting the overall translation output, thereby promoting the occurrence and metastasis of ovarian cancer [86].

### Pancreatic cancer

Pancreatic cancer is the seventh leading cause of cancer death in both males and females worldwide because of its poor prognosis, with a fatality rate of almost 100% [87]. In pancreatic cancer cells, four miRNAs, miR-17-5p, miR-21-5p, miR-200c-3p and miR-let-7a-5p, contain m6A methylation modification sites. Although the expression of these miRNAs does not differ significantly in cancer tissues compared to paired normal tissues, their methylation levels are significantly increased. More importantly, serum levels of methylated miR-17-5p distinguish early pancreatic cancer patients from healthy controls with high sensitivity and specificity [50]. Research showed that the absence of METTL3 increased the sensitivity of pancreatic cancer cells to anticancer drugs, such as gemcitabine, 5-fluorouracil, and cisplatin but had little effect on cell morphology and proliferation. In addition, the authors performed relevant gene ontology, gene/protein interaction, and protein/protein interaction analyses by using the appropriate databases and found that METTL3 was associated with the mitogen-activated protein kinase cascade, ubiquitin-dependent processes, RNA splicing, and regulation of cell processes, suggesting that it plays a functional role in these biological processes [88]. However, ALKBH5 overexpression sensitized pancreatic cancer cells to gemcitabine therapy and inhibited pancreatic cancer tumorigenesis by reducing the levels of m6A-dependent WNT inhibitory factor 1 (WIF-1) and hindering WNT signaling activation [89]. The expression of potassium channel subfamily K member 15 antisense RNA 1 (KCNK15-AS1) in pancreatic cancer tissues was downregulated compared with that in normal pancreatic tissues, and KCNK15-AS1 was shown to promote the migration and invasion of pancreatic cancer cells. In addition, the m6A level in total RNA was significantly increased in cancer cells compared with human pancreatic duct epithelial cells. Moreover, expression of the m6A eraser ALKBH5, which can demethylate KCNK15-AS1 and regulate cell movement mediated by KCNK15-AS1, was downregulated in cancer cells [90]. In addition, upregulation of IGF2BP2 was associated with poor prognosis in patients with pancreatic cancer. IGF2BP2 can be a reader of m6A-modified lncRNA-DANCR and functions to stabilize DANCR, which in turn jointly promotes cancer stemness-like properties and pancreatic cancer pathogenesis [91].

### Nasopharyngeal carcinoma (NPC)

NPC is a malignant head and neck cancer with apparent regional clustering. Approximately 30% of NPC patients develop distant metastasis and/or recurrence [92, 93]. METTL3 was found to inhibit the expression of zinc finger protein 750 (ZNF750) and downregulate the expression of fibroblast growth factor 14 (FGF14) in NPC, which promoted NPC development and progression [94]. In addition, METTL3 activated FAK/PI3K/AKT signaling and promoted the proliferation and invasion of NPC cells by enhancing the stability of lncRNA-FAM225A and promoting the competitive binding of miR-590-3p and miR-1275 [95].

### Lung cancer

Lung cancer remains the leading cause of cancer incidence and mortality worldwide, with 2.1 million new cases and 1.8 million deaths predicted in 2018 [87]. METTL3 relies on YTHDF1/3 and eIF3B to promote yes-associated protein (YAP) mRNA translation and increases YAP mRNA stability by modulating the MALAT1–miR-1914-3p–YAP axis, thereby inducing treatment resistance and metastasis in non-small cell lung cancer (NSCLC) [96]. Additionally, METTL3 plays an important role in TGF-β-induced EMT in lung cancer, mainly by promoting the m6A modification, increasing the total mRNA level, and enhancing the mRNA stability of JUNB, one of the most important transcriptional regulators of EMT [97].

On the other hand, high expression of FTO in lung cancer was found to decrease the m6A levels of ubiquitin-specific peptidase 7 (USP7), improve the mRNA stability of USP7, and promote the occurrence of NSCLC [98]. Furthermore, FTO enhanced the expression of myeloid zinc finger 1 (MZF1) by reducing the m6A level in MZF1 mRNA and enhancing its stability, thus promoting the development of lung cancer [99]. ALKBH5 was found to influence the proliferation and invasion of lung adenocarcinoma cells under intermittent hypoxia (IH) conditions by reducing m6A modification of FOXM1 mRNA and promoting FOXM1 expression [100].

In addition, YTHDF2 was found to be upregulated in lung cancer tissues, to promote cell growth in lung cancer, and to bind directly to the m6A-modified site in the 6-phosphogluconate dehydrogenase (6PGD) 3′ UTR. This event promoted the translation of 6PGD mRNA in lung cancer cells and enhanced flux through the pentose phosphate pathway (PPP), which is critical the promotion of tumor growth [101].

### Hepatocellular carcinoma (HCC)

Liver cancer is the sixth most commonly diagnosed cancer and the fourth leading cause of cancer death worldwide. Types of primary liver cancer include HCC (accounting for 75–85% of cases) and intrahepatic cholangiocarcinoma (ICC) (accounting for 10–15% of cases), as well as other rare types [87].

Zhou et al. analyzed 162 cases of HCC in the TCGA database and the GSE14520 and GSE63898 datasets and found that the combination of METTL3 and YTHDF1 levels could be used as a biological index to reflect the degree of malignancy and evaluate the prognosis of HCC [102]. Additionally, TCGA database analysis showed that the expression of YTHDF1 was significantly upregulated in HCC, and this upregulation was positively correlated with the pathological stage. Kaplan-Meier analysis showed that a low level of YTHDF1 expression was associated with better survival of HCC patients. In addition, GO and KEGG pathway analyses of genes coexpressed with YTHDF1 showed that YTHDF1 played an important role in regulating the cell cycle and metabolism in HCC cells [103]. By analyzing data from the TCGA and GEO databases, Li et al. found that METTL14 may be involved in the malignant progression of HCC by modulating m6A-regulated genes, including cysteine sulfinic acid decarboxylase (CSAD), glutamic-oxaloacetic transaminase 2 (GOT2), and suppressor of cytokine signaling 2 (SOCS2) [104].

Regarding the molecular mechanism, METTL3 increased the m6A level in SOCS2 mRNA, promoted the degradation of SOCS2 mRNA through an m6A-YTHDF2-dependent mechanism, and inhibited the expression of SOCS2 in HCC tissues, thus promoting HCC progression [105]. In HCC cells, METTL3 installed m6A in the coding sequence (CDS) and 3′ UTR regions of a key EMT transcription factor, Snail, thus promoting its translation. Subsequently, interactions involving YTHDF1 and eukaryotic translation elongation factor 2 (eEF-2) enhanced Snail translation, which ultimately promoted the development of HCC [106]. METTL3-mediated m6A modification upregulated the expression of LINC00958 by stabilizing its RNA transcript, which in turn upregulated hepatocarcinoma-derived growth factor (HDGF) expression through sponging miR-3619-5p, thereby promoting HCC lipogenesis and progression [107].

Additionally, KIAA1429 was found to be significantly upregulated in HCC tissues. KIAA1429 induced methylation of the 3′ UTR of GATA binding protein 3 (GATA3) pre-mRNA, resulting in isolation of the RBP HUR and degradation of GATA3 pre-mRNA. Furthermore, lncRNA-GATA3-AS, transcribed from the antisense strand of the GATA3 gene, was shown to serve as a cis-acting element for the preferential interaction of KIAA1429 with GATA3 pre-mRNA [108]. KIAA1429 was shown to promote the migration and invasion of liver cancer cells by increasing the m6A level in the mRNA of inhibitor of DNA binding 2 (ID2) and inhibiting its expression [109]. In addition, WTAP is highly expressed in HCC. m6A modification of WTAP was found to result in posttranscriptional inhibition of ETS1, another mechanism by which the p21/p27-dependent pathway could regulate the cell cycle in HCC cells [110].YTHDF2 had an inhibitory effect on HCC, which inhibited tumor cells and vessels by processing interleukin 11 (IL11) and serpin family E member 2 (SERPINE2) mRNA. However, activation of YTHDF2 in HCC cells was blocked by hypoxia inducible factor 2 subunit A (HIF-2A), indicating that hypoxia increased the epigenetic aggressiveness of HCC cells [111].

In addition, Liu et al. [112] found that enhanced m6A mRNA methylation was an oncogenic mechanism underlying hepatoblastoma (HB). METTL3 was significantly upregulated in HB and promoted its development. Additionally, the authors identified catenin beta 1 (CTNNB1) as a regulator of METTL3-mediated RNA modification in HB.

### Intrahepatic cholangiocarcinoma (ICC)

Rong et al. [113] found that the protein expression of FTO was downregulated in clinical ICC tissues and cell lines. Additionally, FTO expression was found to be correlated with CA19–9 expression, microvessel density (MVD), prognosis, and ICC cell apoptosis and migration. Database mining showed that FTO could regulate integrin signaling, inflammation signaling, epidermal growth factor receptor (EGFR) signaling, angiogenesis, and pyrimidine metabolism pathways.

### Testicular germ cell tumors (TGCTs)

TGCTs account for most (98%) testicular cancers, and the annual number of TGCT cases has more than doubled since the second half of the twentieth century [114]. KIAA1429 and YTHDF3 may play a synergistic role in the establishment of m6A in TGCTs. Their transcriptional levels can accurately distinguish seminomas (SEs) from nonseminomatous tumors (NSTs), possibly providing new candidate biomarkers for patient management [115].

### Melanoma

Melanoma is one of the deadliest and most drug-resistant human cancers in the United States [116]. In melanoma, upregulation of METTL3 expression was found to play a role in the invasion and migration of human melanoma cells by promoting the expression of matrix metallopeptidase 2 (MMP2) [117]. Additionally, FTO was found to promote melanoma growth, while knockout of FTO increased m6A methylation of important genes in malignant melanoma cells, including PD-1, C-X-C motif chemokine receptor 4 (CXCR4), and SRY-Box transcription factor 10 (SOX10), and increased the RNA decay through YTHDF2, which further increased the sensitivity of mouse melanoma cells to interferon gamma (IFNγ) and anti-PD-1 treatment. Collectively, these findings suggest that FTO plays an important role in promoting the occurrence of melanoma and anti-PD-1 resistance [118]. However, decreased m6A levels were found in ocular melanoma samples. In ocular melanoma, YTHDF1 was shown to promote the translation of methylated mRNA of the tumor suppressor HINT2 and inhibit tumor development [119].

### Colorectal cancer (CRC)

CRC is a highly fatal cancer, ranking third in incidence but second in mortality. Its incidence is increasing worldwide [87].METTL3 was found to be highly expressed in CRC and associated with poor prognosis. MeRIP-m6A-seq analysis showed that SOX2 is a downstream gene of METTL3. Methylated SOX2 transcripts, especially the CDS region, can be recognized by IGF2BP2, thus prolonging the half-life of SOX2 mRNA. The expression of SOX2 was found to be positively correlated with that of METTL3 and IGF2BP2 in CRC. Collectively, these findings indicate that METTL3, acting as an oncogene, relies on IGF2BP2 to inhibit SOX2 degradation, thus increasing SOX2 expression [120]. METTL3 downregulation was found to activate p-p38 and p-ERK in CRC; however, p38 or ERK kinase inhibitors significantly reversed the cell migration and invasion induced by METTL3 knockout. METTL3 was also found to inhibit the proliferation, migration, and invasion of CRC cells through the p38/ERK pathway [121]. Furthermore, METTL3 relies on IGF2BP1 to prolong the half-life of chromobox 8 (CBX8) mRNA, which recruits lysine methyltransferase 2B (KMT2B) and Pol-II to the promoter of leucine rich repeat containing G protein-coupled receptor 5 (LGR5) and maintains the H3K4me3 status. Together, these factors promote LGR5 expression, ultimately maintaining the stemness of CRC cells and promoting their chemoresistance [122]. METTL3 can methylate pri-miR-1246 in CRC cells, thus further promoting the maturation of pri-miR-1246, whereas the tumor suppressor gene sprouty related EVH1 domain containing 2 (SPRED2) is a downstream target of miR-1246. Downregulation of SPRED2 further reversed the inhibitory effect on the MAPK pathway [123]. METTL14 was found to be downregulated in CRC tissues and cell lines, with miR-375 as a downstream target. In addition, METTL14 was found to inhibit the growth of CRC cells through the miR-375/YAP1 pathway and to inhibit the migration and invasion of CRC cells through the miR-375/SP1 pathway [124].

YTHDF1 was found to be highly expressed at the mRNA and protein levels in CRC. Downregulation of YTHDF1 expression significantly inhibited the tumorigenicity of CRC cells and the growth of mouse xenografts. In addition, silencing YTHDF1 significantly inhibited the activity of the WNT/β-catenin pathway in CRC cells [125]. In addition, Wu et al. found that m6A-induced lncRNA-RP11 promoted the proliferation of CRC cells by upregulating the expression of zinc finger E-Box binding homeobox (ZEB). Thus, YTHDF1 plays an important role in the progression of CRC [126].lncRNA-GAS5 binds directly to the WW domain of YAP in CRC and promotes its phosphorylation and ubiquitin-mediated degradation, thereby weakening YAP-mediated YTHDF3 transcription. YTHDF3 was shown to bind reversibly and selectively to m6A-methylated GAS5, triggering its decay and forming a GAS5-YAP-YTHDF3 negative feedback loop to promote the development of CRC [127].

### Bladder cancer

Bladder cancer is the 10th most common cancer worldwide [87]. The expression of METTL3 in human bladder cancer was shown to be significantly upregulated. AF4/FMR2 family member 4 (AFF4), the NF-κB pathway, and MYC were further identified as direct targets of METTL3-mediated m6A modification. In addition, AFF4 promoted the expression of the MYC promoter by binding to it, suggesting that there is a multilevel regulatory network downstream of METTL3 [128]. Furthermore, Han et al. found that METTL3 promoted the maturation of pri-miR221/222 and reduced the expression of phosphatase and tensin homolog (PTEN), which resulted in the proliferation of bladder cancer cells. This study confirmed the carcinogenic role of METTL3 in bladder cancer [129].

### Prostate cancer

Prostate cancer is the second most common cancer and the fifth leading cause of cancer death in men [87]. Cai et al. found that METTL3 was highly expressed in prostate cancer, where it promoted m6A modification and expression of GLI family zinc finger 1 (GLI1), an important component of the Hedgehog pathway, and regulated the Hedgehog pathway to promote prostate cancer development [130].

### Breast cancer

Breast cancer is the most common cancer among women in most countries, and its incidence has increased in many South American, African, and Asian countries over the past few decades [87]. METTL3 was found to be upregulated in breast cancer tissues and cells, where it promoted m6A modification of the 3′ UTR of B-cell/lymphoma 2 (BCL-2) mRNA. This modification resulted in upregulation of BCL-2 expression, thereby promoting cell proliferation, inhibiting apoptosis, and promoting tumor growth [131]. Additionally, FTO was found to be upregulated in breast cancer tissues. The apoptosis-promoting gene BCL2 interacting protein 3 (BNIP3) is the downstream target of FTO-mediated m6A modifications. FTO mediates m6A demethylation in the 3′ UTR of BNIP3 mRNA and induces its degradation via a YTHDF2-dependent mechanism [132].

### Renal cancer

Renal cell carcinoma (RCC) is the most lethal of all malignant tumors of the urinary system [87]. RCC is substantially resistant to radiotherapy and chemotherapy [133]. Zhou et al. found that alterations in m6A-regulating factors in clear cell renal cell carcinoma (ccRCC) led to a significant association between the level of m6A and the worsening of clinical parameters, including survival. The VHL-HIF-METTL3/14 pathway may be involved in the regulation of m6A in ccRCC, and the PI3K-mTOR and p53 signaling pathways may be downstream targets of m6A in ccRCC [134]. Additionally, ATP-P2RX6 may promote the migration and invasion of RCC cells by regulating the Ca-mediated p-ERK1/2/MMP9 signaling pathway. In addition, METTL14 was found to be involved in m6A modification and downregulation of purinergic receptor P2X 6 (P2RX6) protein translation in RCC [135]. In addition, the protein expression levels of ALKBH5 and FTO in renal cancer tissues were significantly lower than those in normal kidney tissues and other tumor tissues. Moreover, the decrease in ALKBH5 and FTO mRNA levels was associated with a decrease in total and tumor-specific survival times after nephrectomy [136].

### Gastric cancer (GC)

GC is the fifth most commonly diagnosed cancer worldwide and the third leading cause of cancer death [87]. A study of the TCGA database found that a low m6A signal predicted poor clinicopathological features of GC, while a decrease in m6A RNA methylation activated the carcinogenic WNT/PI3K-AKT signaling pathway and promoted the development of GC [137]. Regarding the molecular mechanism, METTL3 is upregulated in GC and predicts a poor prognosis. m6A modification of zinc finger MYM-Type containing 1 (ZMYM1) mRNA by METTL3 relies on a HUR-dependent pathway to enhance its stability. ZMYM1 promotes the EMT program and cell migration by recruiting the CTBP/LSD1/COREST complex to bind to the E-cadherin promoter and mediate inhibition of the E-cadherin promoter [138].

Additionally, p300 mediates H3K27 acetylation-induced activation of the METTL3 promoter to induce METTL3 transcription, whereas METTL3 catalyzes m6A modification of HDGF mRNA. IGF2BP3 was shown to directly recognize and bind to the m6A site on HDGF mRNA, thereby enhancing its stability. Subsequently, secretory HDGF promoted tumor angiogenesis, and nuclear HDGF activated insulin-responsive glucose transporter 4 (GLUT4) and enolase 2 (ENO2) expression, which in turn promoted glycolysis in GC cells and ultimately promoted cell growth and liver metastasis [139].Lin et al. [140] found that downregulation of METTL3 inhibited the proliferation and migration of human GC cells and inactivated the AKT signaling pathway, a finding that was supported by Liu et al. [141].Low protein expression levels of FTO were correlated with reduced overall survival times in GC patients. The expression of the demethylase gene FTO in GC patients had obvious prognostic value, suggesting that it may play an important role in the progression and metastasis of GC [142].

### Acute myeloid leukemia (AML)

AML is one of the most common and lethal hematopoietic malignancies, with different genetic and molecular abnormalities [143, 144]. The mRNA and protein expression of METTL3 were found to be upregulated in AML cells. Deletion of METTL3 in a human myeloid leukemia cell line was shown to induce the differentiation and apoptosis of cells in recipient mice and delay the occurrence of leukemia. In addition, m6A was shown to promote the translation of c-MYC, BCL2, and PTEN mRNA in human myeloid leukemia cells [145]. In immunodeficient mice, downregulation of METTL3 led to cell cycle arrest, leukemia cell differentiation, and the inability to establish leukemia. Moreover, METTL3 was independent of METTL14 binding to chromatin and was located at the transcription start site (TSS) of active genes. Promoter-bound METTL3 induced m6A modification within the coding region of the related mRNA transcripts and enhanced the translation of these transcripts by removing the stalled ribosomes [146]. Both knockout and overexpression of the METTL3 protein led to upregulation of WTAP protein expression, suggesting that METTL3 levels play a key role in WTAP protein homeostasis. However, upregulation of WTAP alone was insufficient to promote cell proliferation in the absence of functional METTL3. Therefore, the carcinogenic function of WTAP is strictly related to functional m6A methylation complexes [147].

Additionally, METTL14 was found to be highly expressed in normal hematopoietic stem/progenitor cells (HSPCs) and AML cells carrying t (11q23), t (15;17), or t (8;21) translocations. The expression of METTL14 was downregulated during myeloid differentiation. Silencing of METTL14 promoted myeloid terminal differentiation of normal HSPCs and AML cells and inhibited the survival and proliferation of AML cells. Mechanistically, METTL14 was shown to modulate its target mRNAs, such as MYB and MYC, through m6A modification, which was negatively regulated by SPI1. Collectively, these findings may help reveal the role of the SPI1-METTL14-MYB/MYC signaling axis in hematopoiesis and leukemia [148].WTAP was found to be upregulated in AML cells and to play an important role in the abnormal proliferation of leukemia cells and the inhibition of their differentiation, suggesting WTAP as a potential new therapeutic target for AML [149].

FTO plays an important carcinogenic role in AML. FTO was found to be upregulated in AML with t (11q23)/MLL rearrangements or t (15;17)/PML-RARA, FLT3-ITD, and/or NPM1 mutations. FTO was found to promote leukemia oncogene-mediated cell transformation and leukemia, inhibit the differentiation of AML cells induced by all-trans retinoic acid (ATRA), and regulate the expression of its target genes, such as ankyrin repeat and SOCS box containing 2 (ASB2) and retinoic acid receptor alpha (RARA), by decreasing the m6A level in mRNA transcripts [150]. R-2-hydroxyglutarate (R-2HG) has a wide range of antileukemia activities in vitro and in vivo by inhibiting the proliferation/survival of leukemic cells and promoting cell cycle arrest and apoptosis. Regarding the biological mechanism, R-2HG was shown to inhibit FTO activity, thus increasing the m6A mRNA modification in R-2HG-sensitive leukemia cells, reducing the stability of MYC/CEBPA transcripts, and inhibiting the related pathways. In conclusion, R-2HG can inhibit the proliferation/survival of cancer cells with high FTO expression levels by targeting the FTO/m6A/MYC/CEBPA signaling cascade [151].

Finally, overexpression of YTHDF2 in AML reduced the half-life of various m6A transcripts, including those of TNF receptor superfamily member 2 (TNFRSF2), which contributes to the overall function of leukemic stem cells (LSCs). Additionally, deletion of YTHDF2 enhanced the activity of hematopoietic stem cells (HSCs). Therefore, the authors suggested that YTHDF2 may be a unique therapeutic target, as its inhibitory effect can selectively inhibit LSCs while promoting the proliferation of HSCs [152].

### Osteosarcoma

Osteosarcoma is one of the most common cancers, with a high mortality rate and heterogeneous presentation [153, 154]. The expression of METTL3 in human osteosarcoma tissue and osteosarcoma cell lines was found to be significantly upregulated, a characteristic that was related to osteosarcoma cell proliferation, migration, and invasion. Further mechanistic analysis showed that silencing METTL3 decreased the mRNA level of lymphoid enhancer-binding factor 1 (LEF1) and then inhibited the activity of the WNT/β-catenin signaling pathway. Thus, METTL3 promotes the growth of osteosarcoma cells by regulating the m6A level of LEF1 and activating the WNT/β-catenin signaling pathway [155]. Additionally, ALKBH5 was shown to reduce m6A modification of lncRNA-PVT1 and inhibit the binding of the reader protein YTHDF2 to PVT1, thereby inhibiting PVT1 degradation. Thus, PVT1 plays a carcinogenic role in osteosarcoma [156].

## Conclusion

In this review, we summarized the physiological functions of m6A-related regulators, current methods for detecting m6A modification, m6A-related databases and, most importantly, the potential role of m6A in cancer. Writers can catalyze m6A installation on RNA, while erasers can remove these modifications. Finally, recognition of m6A methylation by readers affects mRNA splicing, export, degradation, translation, and other biological processes. In addition, m6A methylation and its related regulatory factors can affect the processing of miRNAs and the biological function of lncRNAs and can even promote the translation of circRNAs. Thus, m6A and its related regulatory factors play various important roles in cancers.

Interestingly, m6A seems to act as a double-edgedsword in cancer. Some genes can promote tumor development after methylation, while others, after removal of methylation, can promote tumor development. For example, in CRC, SOX2 plays a cancer-promoting role through METTL3-catalyzed methylation [120], while in breast cancer, BNIP3 plays a cancer-promoting role through FTO-catalyzed demethylation [132]. Additionally, the same m6A-associated regulator can perform biological functions through different target genes in the same cancer. For example, in GC, METTL3 can promote cancer by catalyzing the methylation of either ZMYM1 [138] or HDGF [139]. In addition, studies have reported conflicting findings in the same type of cancer; for example, in liver cancer, METTL3 and METTL14 play completely opposite roles in tumor development [105, 157]. Collectively, these findings indicate that complex regulatory networks of m6A methylation are active in different cancers, awaiting further exploration. In addition, Lin et al. found that METTL3 plays a procancer role in lung cancer [158], while Du et al. found that METTL3 is downregulated in NSCLC [159]. The above discrepancy may be because the case sample size was insufficient in these studies. In addition, some researchers have focused on targeted cancer therapies related to m6A, such as treatment with R-2HG to increase m6A mRNA modification in R-2HG-sensitive leukemia cells by inhibiting FTO activity, thus generating antileukemia effects [151]. However, a limited number of studies have focused on such targeted cancer therapies. Finally, research on the role of m6A in noncoding RNAs in cancer remains rare, with only a few reports in cervical, pancreatic, nasopharyngeal, colorectal, and osteosarcoma.

The rapidly developing study of m6A modifications marks a great advance in molecular biology. Epitranscriptomics, an important subfield of epigenetics, can help reveal the biological mechanisms underlying cancer development and may provide new targets for cancer treatment. A full understanding of the mechanism underlying m6A modification is distant. However, we believe that future research on m6A modifications will focus on the following topics: first, construction of a complex regulatory network model of m6A and its associated modifiers in a single cancer; second, expansion of the clinical case sample size and screening factors that can be used as predictors for early diagnosis and prognosis; and third, development of m6A-related targeted cancer therapies to provide new potential options for cancer treatment.

## Data Availability

Not applicable.

## Abbreviations

m6A: N6-methyladenine
FTO: fat mass and obesity-associated protein
DM2: type 2 diabetes mellitus
MTC: methyltransferase complex
METTL3: Methyltransferase-like protein 3
SAM: S-adenosyl methionine
METTL14: Methyltransferase-like protein 14
SAH: T-adenosyl homocysteine
WTAP: WT1-associated protein
RBM15: RNA-binding motif protein 15
RBM15B: RNA-binding motif protein 15B
VIRMA/KIAA1429: Vir-like m6A methyltransferase associated
CPSF5: cleavage and polyadenylation specificity factor subunit 5
CPSF6: cleavage and polyadenylation specificity factor subunit 6
ZC3H13: zinc finger CCCH-type containing 13
LC: low-complexity
METTL16: Methyltransferase-like protein 16
LARP7: La-related protein 7
MEPCE: methylphosphate capping enzyme
ALKBH5: AlkB homolog 5
hm6A: N6-hydroxymethyladenosine
f6A: N6-formyladenosine
ALKBH3: AlkB homologue 3
YTHDF1: YTH domain family protein 1
YTHDF2: YTH domain family protein 2
YTHDF3: YTH domain family protein 3
YTHDC1: YTH domain containing 1
YTHDC2: YTH domain containing 2
CNOT1: CCR4-NOT transcription complex subunit 1
eIF3: eukaryotic translation initiation factor 3
eIF4E: eukaryotic translation initiation factor 4E
eIF4G: eukaryotic translation initiation factor 4G
PABP: poly(A) binding protein
SRSF3: serine- and arginine-rich splicing factor 3
SRSF10: serine- and arginine-rich splicing factor 10
NXF1: nuclear RNA export factor 1
TREX: three prime repair exonuclease
MEIOC: meiosis-specific coiled-coil domain
XRN1: 5′-3′ exoribonuclease 1
HNRNP: heterogeneous nuclear ribonucleoprotein
DROSHA: drosha ribonuclease III
DGCR8: DiGeorge syndrome critical region 8
IGF2BPs: Insulin-like growth factor 2 mRNA-binding proteins
miRNAs: microRNAs
lncRNAs: long-noncoding RNAs
circRNAs: circular RNAs
GBM: glioblastoma
GSCs: Glioblastoma stem cells
SOX2: SRY-box transcription factor 2
ADAR: adenosine deaminase RNA specific
APOBEC3A: apolipoprotein B mRNA editing enzyme catalytic subunit 3A
ADAM19: ADAM metallopeptidase domain 19
CSCC: cervical squamous cell carcinoma
E2F1: E2F transcription factor 1
WIF-1: WNT inhibitory factor 1
KCNK15-AS1: potassium channel subfamily K member 15 antisense RNA 1
NPC: Nasopharyngeal carcinoma
ZNF750: zinc finger protein 750
FGF14: fibroblast growth factor 14
YAP: yes associated protein
NSCLC: non-small cell lung cancer
EMT: epithelial-mesenchymal transition
USP7: ubiquitin specific peptidase 7
MZF1: myeloid zinc finger 1
6PGD: 6-phosphate gluconate dehydrogenase
PPP: pentose phosphate pathway
HCC: Hepatocellular carcinoma
ICC: intrahepatic cholangiocarcinoma
CSAD: cysteine sulfinic acid decarboxylase
GOT2: glutamic-oxaloacetic transaminase 2
SOCS2: suppressor of cytokine signaling 2
eEF-2: eukaryotic translation elongation factor 2
HDGF: hepatocarcinoma-derived growth factor
GATA3: GATA binding protein 3
ID2: inhibitor of DNA binding 2
IL11: interleukin 11
SERPINE2: serpin family E member 2
HIF-2A: hypoxia inducible factor 2 subunit A
HB: hepatoblastoma
CTNNB1: catenin beta 1
EGFR: epidermal growth factor receptor
TGCT: Testicular germ cell tumors
MMP2: matrix metallopeptidase 2
CXCR4: C-X-C motif chemokine receptor 4
SOX10: SRY-Box transcription factor 10
CRC: Colorectal cancer
CDS: coding sequence
CBX8: chromobox 8
KMT2B: lysine methyltransferase 2B
LGR5: leucine rich repeat containing G protein-coupled receptor 5
SPRED2: sprouty related EVH1 domain containing 2
ZEB: zinc finger E-Box binding homeobox
AFF4: AF4/FMR2 family members 4
PTEN: phosphatase and tensin homolog
GLI1: GLI family zinc finger 1
BCL-2: B-cell cll/lymphoma 2
BNIP3: BCL2 interacting protein 3
BCSCs: breast cancer stem cells
ZNF217: zinc finger protein 217
KLF4: kruppel like factor 4
RCC: Renal cell carcinoma
ccRCC: clear cell renal cell carcinoma
P2RX6: purinergic receptor P2X 6
GC: Gastric cancer
ZMYM1: zinc finger MYM-Type containing 1
GLUT4: insulin-responsive glucose transporter 4
ENO2: enolase 2
AML: Acute myeloid leukemia
TSS: transcriptional start site
HSPCs: hematopoietic stem/progenitor cells
ATRA: all-trans-retinoic acid
ASB2): ankyrin repeat and SOCS box containing 2
RARA: retinoic acid receptor alpha
R-2HG: R-2-hydroxyglutarate
TNFRSF2: TNF receptor superfamily member 2
LSCs: leukemic stem cells
HSCs: hematopoietic stem cells
LEF1: lymphoid enhancer-binding factor 1
